# Retrogradely Transportable Lentivirus Tracers for Mapping Spinal Cord Locomotor Circuits

**DOI:** 10.3389/fncir.2018.00060

**Published:** 2018-07-25

**Authors:** Imran S. Sheikh, Kathleen M. Keefe, Noelle A. Sterling, Ian P. Junker, Chidubem I. Eneanya, Yingpeng Liu, Xiao-Qing Tang, George M. Smith

**Affiliations:** Department of Neuroscience, Shriners Hospitals Pediatric Research Center, Center for Neural Rehabilitation and Repair, Lewis Katz School of Medicine, Temple University, Philadelphia, PA, United States

**Keywords:** retrograde tracing, lentivirus, propriospinal, corticospinal, rubrospinal, reticulospinal, spinal cord, spontaneous recovery

## Abstract

Retrograde tracing is a key facet of neuroanatomical studies involving long distance projection neurons. Previous groups have utilized a variety of tools ranging from classical chemical tracers to newer methods employing viruses for gene delivery. Here, we highlight the usage of a lentivirus that permits highly efficient retrograde transport (HiRet) from synaptic terminals within the cervical and lumbar enlargements of the spinal cord. By injecting HiRet, we can clearly identify supraspinal and propriospinal circuits innervating motor neuron pools relating to forelimb and hindlimb function. We observed robust labeling of propriospinal neurons, including high fidelity details of dendritic arbors and axon terminals seldom seen with chemical tracers. In addition, we examine changes in interneuronal circuits occurring after a thoracic contusion, highlighting populations that potentially contribute to spontaneous behavioral recovery in this lesion model. Our study demonstrates that the HiRet lentivirus is a unique tool for examining neuronal circuitry within the brain and spinal cord.

## Introduction

Early mapping studies of neuroanatomical pathways of the central and peripheral nervous system utilized tracing of neurons and axons with chemical tracers. Markers such as biotinylated dextran amines (BDAs) (Veenman et al., 1992), cholera toxin beta subunit (Trojanowski et al., 1982; Luppi et al., 1990), fluorogold (Schmued and Fallon, 1986), microbeads (Katz et al., 1984; Katz and Iarovici, 1990) and *Phaseolus vulgaris*-leucoagglutinin (Gerfen and Sawchenko, 1984) provide a clear and distinct labeling of neuronal morphology including somas, dendrites, and axons. Each of these methods permit investigators to examine different populations of neurons based on injection volume, sensitivity of labeling, diffusion, and resistance to fading. Depending on the type of tracer used, visualization of neuronal populations can be enhanced through immunohistochemistry.

Classical tracers such as BDA and fluorogold are considered gold standard for many experiments, but they do have certain disadvantages. BDA can be taken up by damaged axons in the injection area, or by axons in passage in the white matter surrounding an injection site (Brandt and Apkarian, 1992; Reiner et al., 2000). This may lead to incorrect interpretations of neuronal numbers or connecting pathways and in studies examining axon regeneration, BDA can be absorbed by damaged or severed axons and mistaken for regenerating fibers (Steward et al., 2007). Lastly, use of these tracers does not provide information regarding synaptic connectivity of a set population of neurons.

Viral vectors are important tools in neurological studies because of their unique ability to transduce specific neuronal populations *in vivo* and express select transgenes within the cell somas or axon terminals. Several neurotropic viruses travel via retrograde transport from axon terminals, allowing analysis of long-projection neurons, and introducing greater neuronal specificity because of the method of uptake. Examples of viruses that exhibit retrograde labeling include adeno-associated (rAAV-retro), rabies (RV) and pseudorabies (PRV) (Baer et al., 1965; Wickersham et al., 2007; Callaway and Luo, 2015; Tervo et al., 2016). RV can quickly transport along multiple neuronal synapses, but has high toxicity, making time sensitive analysis for tract tracing studies crucial (Coulon et al., 1989; Kelly and Strick, 2000; Reardon et al., 2016). The Bartha strain of PRV is a popular method to retrogradely map neuronal circuits in both the brain and spinal cord, but like RV, has similar issues regarding neurovirulence and toxicity (see Aston-Jones and Card, 2000 for review). Finally, AAV – including retrogradely transportable forms – can be taken up by axons in passage, rendering circuit mapping difficult to interpret (Tervo et al., 2016).

Lentiviral vectors based on the human immunodeficiency virus type-1 (HIV-1) provide stable, long-term expression of transgenes in transduced neuronal populations and they are widely used in studies involving gene therapy (Wang et al., 1999; Pawliuk et al., 2001; Lo Bianco et al., 2004; Adjali et al., 2005; Malik et al., 2005; Brown et al., 2007). Such vectors offer similar advantages for labeling neuronal populations distant from the injection site. However, most HIV-1 lentiviral vectors are packaged with vesicular stomatitis viral glycoprotein (VSV-G), which, though easy to concentrate and create a high-titer product, can limit retrograde transport of the transgene, and is toxic to some mammalian cells if constitutively expressed (Burns et al., 1993). VSV-G pseudotyped vectors may also trigger an immune response directed against the envelope protein when used *in vivo* (DePolo et al., 2000; Higashikawa and Chang, 2001). The shortcomings of VSV-G have been alleviated in some cases via chemical modification of the envelope glycoprotein. For example, immune system reaction can be tempered, and transduction efficiency increased when VSV-G is conjugated with poly-ethylene glycol (PEG) (Croyle et al., 2004). To improve retrograde transport, Kato et al. (2011b) and Hirano et al. (2013) have pseudotyped lentiviral vectors with a fusion envelope glycoprotein of rabies virus (FuG-B) to produce a highly efficient retrograde transport vector termed HiRet. HiRet lentivirus has been used to specifically target C3-C4 propriospinal (PN) neurons in the macaque in combination with adeno-associated virus 2 (AAV2) expressing TetOn for reversible synaptic silencing experiments (Kinoshita et al., 2012; Tohyama et al., 2017).

This vector seems to be an excellent choice for mapping central nervous system pathways because of the advantages outlined above: it does not negatively impact the survival or function of infected neurons, and it is stable, long-lasting and easily detectable. Although HiRet is not entirely specific to neurons, as it also labels astrocytes at the injection site, it does show selective uptake at the synapse of neurons and does not spread into injured axons or unrelated cells regardless of the dosage or time course of the study (Ugolini, 1995; Morcuende et al., 2002). In addition, coupling HiRet lentivirus containing a tetracycline-inducible promoter to drive transgene expression with a second virus, AAV2-TetOn, allows very tight retrograde labeling of specific neuronal populations. Here, we use constitutively active HiRet-GFP to map supraspinal and propriospinal connections terminating in the cervical or lumbar regions of the rat spinal cord. We highlight the advantages this lentivirus provides for mapping studies in the central nervous system and in the context of thoracic spinal cord injury.

## Materials and Methods

### Animals

All surgical and animal care protocols were approved by the Temple University School of Medicine’s Institutional Animal Care and Use Committee and performed per the National Institutes of Health *Guide for the Care and Use of Laboratory Animals*. Female Sprague-Dawley rats (65–75 days, 200–224 g; Harlan Laboratories) were housed two per cage, on a 12-h light-dark cycle with food and water provided *ad libitum*. Animals were allowed 7 days of acclimatization prior to any experimental procedure. At the time of surgery animals ranged in weight from 225 to 250 g. Female rats were used primarily for spinal cord contusion studies which require manual bladder expression twice daily.

### Viral Vectors

The HiRet lentiviral vector is an HIV-1 pseudotype that allows retrograde transport and labeling of neurons. For retrograde tract tracing purposes, we generated HiRet-GFP to label long descending projection neurons to the cervical and lumbar enlargement. HiRet-GFP was constructed by packaging a GFP plasmid (pCSC-SP-PW-GFP, Addgene plasmid # 12337, a gift from Inder Verma) (Marr et al., 2004) with rabies virus fusion envelope glycoprotein G (FuG-B; provided by Dr. Kobayashi) and plasmids pMbl and pRev. A plasmid, pLV-TRE-GFP (also provided by Dr. Kobayashi) was packaged in a similar manner to produce HiRet-TRE-GFP for specific labeling of PNs. Rabies virus glycoprotein allows HiRet to transduce synapses and retrogradely transport with high efficiency into neuronal somas. Plasmids were transfected into HEK293T cells using the CaPO_4_ method and allowed to incubate overnight. Purified virus was suspended in Tris buffer containing rat albumin and mannitol. Lentiviral titers were determined using a P24 HIV-1 ELISA kit and were around 2 × 10^7^ TU/mL infectivity prior to storage in -80°C.

AAV2-CMV-rtTAV16 (AAV-TetOn) was used in combination with HiRet-TRE-GFP to unilaterally label C3-C4 propriospinal interneurons. The plasmid pAM-rtTAV16 was generously provided by Dr. Kobayashi and was packaged using the helper-free method as reported previously (Ayuso et al., 2010; Liu et al., 2014). In brief, HEK293T cells at 70–80% confluency were transfected with two packaging plasmids, one carrying AAV *rep* and *cap*, the other with AAV helper functions and the transgene using a polyethylenimine method (PEI) (polyethylenimine, linear, MW 25k, Warrington, PA, United States). Three days post transfection, cell supernatant and lysates were harvested. 40% PEG 8000 was added to precipitate crude virus for 2 h. AAV samples were double-ultracentrifuged in a cesium chloride gradient with isolated viral fractions dialyzed in 0.1 M PBS/0.5% Sorbitol overnight (Ayuso et al., 2010; Liu et al., 2014). Purified fractions were added to HEK293T cells previously transduced with HiRet-TRE-GFP to verify function. Quantitative real-time PCR of purified viral fractions was done to determine viral titer. Viral titer of AAV2 was 1.2 × 10^13^ GC/mL.

### Surgical Procedures

Animals were anesthetized with a ketamine (67 mg/kg)/xylazine (6.7 mg/kg, i.p.) mixture. A 0.5 cm incision is made through the dorsal skin above vertebra C4-C6. The skin is retracted to expose the underlying muscle. The exposed spinotrapezius muscle is cut along the midline with scissors, then spread with a small Alm retractor. The muscles are freed from the cervical vertebrae with scissors. The first thoracic vertebrae has a very prominent dorsal spinal process for easy identification The right dorsal vertebral arch and the dorsal spinous process are carefully removed from vertebra C6-T1, so not to damage the spinal cord. Precise injections into the intermediate zone of the spinal cord was performed according to our previously established protocols (Romero et al., 2000, 2001; Tang et al., 2004, 2007; Kelamangalath et al., 2015; Liu et al., 2016) This was done using a beveled glass micropipette pulled to a diameter of 30–40 μm connected to a nanoliter injector (Nanoject, Drummond Scientific). Coordinates were determined from our extensive experience with spinal cord injections and using a micromanipulator (Narishige International) for precise measurements. All coordinates were taken from the midline or surface of the spinal cord. The midline was determined either by the position of the posterior spinal artery or the midpoint between the entry zone of the dorsal roots. Unilateral injections (right side) of HiRet-GFP (1 μL/injection; six injection sites 0.5 mm apart) were made at respective spinal levels targeting the intermediate gray matter; 0.8 mm lateral to the posterior sulcus and 1.5 mm from the dorsal surface of the spinal cord, based on ours and others previously published reports (Watson et al., 2009; Filli et al., 2014). For lumbar injections, musculature was cleared from the T11-T13 vertebral bodies, and hemi-laminectomies were performed to expose the L1-L4 spinal cord. Vertebral positions were determined by identification of the last rib at T13 and counting vertebral segments. It is well established that the spinal cord does not extend the entire length of the vertebral column and L1-L4 spinal cord lays directly under the T12-L1 vertebrae (Gelderd and Chopin, 1977) HiRet-GFP was injected with a beveled pulled glass needle with an aperture of 30–40 μm into six evenly spaced sites (1 mm) along this length. Injections were made lateral to the midline at 0.8 mm. Virus was injected at three separate depths of 0.5 mm, 1.0 mm, and 1.5 mm, for a total volume of 2 μl per site (0.7 μl at each depth). All injections were done using a nanoliter injector (Nanoject, Drummond Scientific) attached to a micromanipulator (Narishige International) for precise measurements. After tracer injections, all animals were maintained for an additional 4 weeks before euthanasia.

### Contusion Injuries

An infinite horizons impactor was used to deliver a severe contusion (200 kilodynes) to the T10 spinal cord. See above injection protocol for how spinal cord level was identified. This injury affects hindlimb locomotion, which we measure by observing the animals’ locomoting across a flat plane, and analyzing the details of stepping, weight bearing, correct foot placement, posture, and coordination with the BBB scale (Basso et al., 1995). We observed a plateau of spontaneous behavioral recovery at about 4 weeks, and therefore chose this time point to inject constitutively active HiRet-GFP tracer into the spinal cord, to map reformed connections. HiRet-GFP was injected into the L1-L4 cord, below the lesion. The intermediate gray matter was targeted to infect a dense population of thoracic propriospinal neurons (TPNs) and long descending propriospinal neurons (LDPNs) (see Flynn et al., 2011 for review). Since bladders need to be expressed twice a day for the first 1–2 weeks after contusion injury, female rats were used in this study. To allow the rats to recovery for 4 weeks to promote any spontaneous locomotor recovery before injections of HiRet-GFP tracer. We then waited another 4 weeks to insure good expression of the tracers before euthanizing the rats.

### Tissue Processing and Histology

At the completion of each experiment (4–6 weeks after the last injections), all animals were euthanized by injection of Fatal-Plus (Dearborn, MI, United States) and perfused with saline (0.9% NaCl) followed by 4% paraformaldehyde (PFA) in 0.1 M phosphate buffer (pH 7.5). The brain and spinal cord were promptly dissected. Spinal cord samples were dissected with dorsal roots intact for identification of each spinal level. Samples were post-fixed in 4% PFA overnight at 4°C. Tissue samples were cut into segments according to gross anatomical landmarks. The spinal cord was cut into segments in accordance to its level by counting the dorsal roots from well-established landmarks, either the first spinal nerve (C1) or the exit of the T13 dorsal root out of the vertebrae with the last identifiable rib. Brain and brainstem blocks were carefully identified based on the rat brain atlas (Paxinos and Charles, 2007) and cut into regions using a 1 mm rat brain matrix (Ted Pella, Reading, CA, United States). The medulla was isolated by first placing a single edge razor blade through the slot closest to back of the cerebellum, and the next blade 6 mm rostral. The pons was isolated from the next 3 mm slice, extending to about the middle of the inferior colliculus. The midbrain was the next rostral 3 mm section extending to just anterior to the superior colliculus. All slices were then transferred to a series of graded sucrose (10%, 20%, and 30%) where they remained for the following 24 h or until the brain section sunk to the bottom of the container. For stereology, tissue blocks were serially sectioned, sagittally at 40 μm intervals using a Leica CM3050S cryostat. Here individual sections were collected on every fifth slide, so that each slide contained the representative serial sections separated by 200 μm. These sections were then stored at -20°C until processed for histology.

### Immunofluorescence

To amplify the GFP signal, slides containing every fifth section were permeabilized in 0.3% Triton X-100 with 5% normal donkey serum to block non-specific binding sites. Samples were then incubated with chicken-anti-GFP primary antibody (1:1000; #GFP-1020; Aves Labs Inc., Tigard, OR, United States) overnight at 4°C. The next day, samples were incubated with donkey-anti-chicken-AlexaFluor 488 secondary (1:400, Jackson ImmunoResearch Laboratories, Inc., West Grove, PA, United States), mounted and photographed using AxioVision software (Carl Zeiss Microscopy, Thornwood, NY, United States).

### Stereology

Unbiased stereological estimates of GFP-positive neurons were performed as previously described (West et al., 1991). Systematic random sampling of GFP-positive neurons were acquired using Stereo Investigator (MicroBrightField, Inc., Williston, VT, United States). Spinal cord segments were identified prior to tissue cryosectioning and the contours between gray and white matter identified by differential phase or dark field imaging. The laminae of Rexed were identified as illustrated in our previous publications (Romero et al., 2000, 2001; Tang et al., 2007) and verified based on gray matter contour from a rat spinal cord atlas (Watson et al., 2009). Boundaries were drawn around the entire gray matter of C3-C4, C4-C7 or T5-T8. Ipsilateral and contralateral gray matter was generally determined by density of GFP signal in axons in the corresponding white matter.

Brain sections were processed as follows: midbrain sections identified containing the red nucleus were quantified from bregma -5.20 mm to -6.70 mm (Paxinos and Charles, 2007). The red nucleus was identified within this region as an almost circular cluster of neurons ventrolateral to the periaqueductal gray and bounded medially by the medial raphe and laterally by the medial lemniscus as describe in our earlier studies (Wang et al., 2011; Liu et al., 2014). Neurons within the caudal pontine reticular formation (PRF) were quantified from bregma -9.10 mm to -10.3 mm. The boundaries shown in **Figure 4** were identified to be dorsal to the ventral pontine nuclei, lateral to the raphe nuclei, and medial to the subcoeruleus nuclear cluster (Paxinos and Charles, 2007). Neurons within the gigantocellular region of the medullary reticular formation (MRF) were quantified from sections identified from bregma -11.0 mm to -12.5 mm. These nuclei were lateral to the medial lemniscus/spinotectal tract, dorsal to the pyramidal tract and medial to the intermediate reticular formation (Paxinos and Charles, 2007). A 350 × 350 μm random sampling grid was placed on all regions of interest for spinal cord gray matter, red nucleus and brainstem reticular areas. Guard zone height was 5 μm (top and bottom) with a sampling brick depth of 20 μm for spinal cord or 40 μm for brain areas. Z-stacks were taken at each region of interest with a 40× objective using a Leica fluorescent microscope. GFP-positive neurons were marked if inside a counting frame (75 μm × 75 μm for spinal cord; 100 μm × 100 μm for brain) using the optical fractionator workflow. Total estimates of neurons labeled in the C3-C4, C4-C7, T5-T8, red nucleus and reticular formation (pontine and medullary) ipsilateral and contralateral to the injection site were reported.

### Statistical Analysis

All statistical analysis was performed using GraphPad Prism 6 (GraphPad Software, Inc., La Jolla, CA, United States). Statistical evaluations of stereological data were analyzed by two-way ANOVA followed by a Sidak *post hoc* test for statistical significance or an unpaired two-tailed *t*-test wherever mentioned. All morphological analysis is represented as mean ± SD.

## Results

**FIGURE 1:**
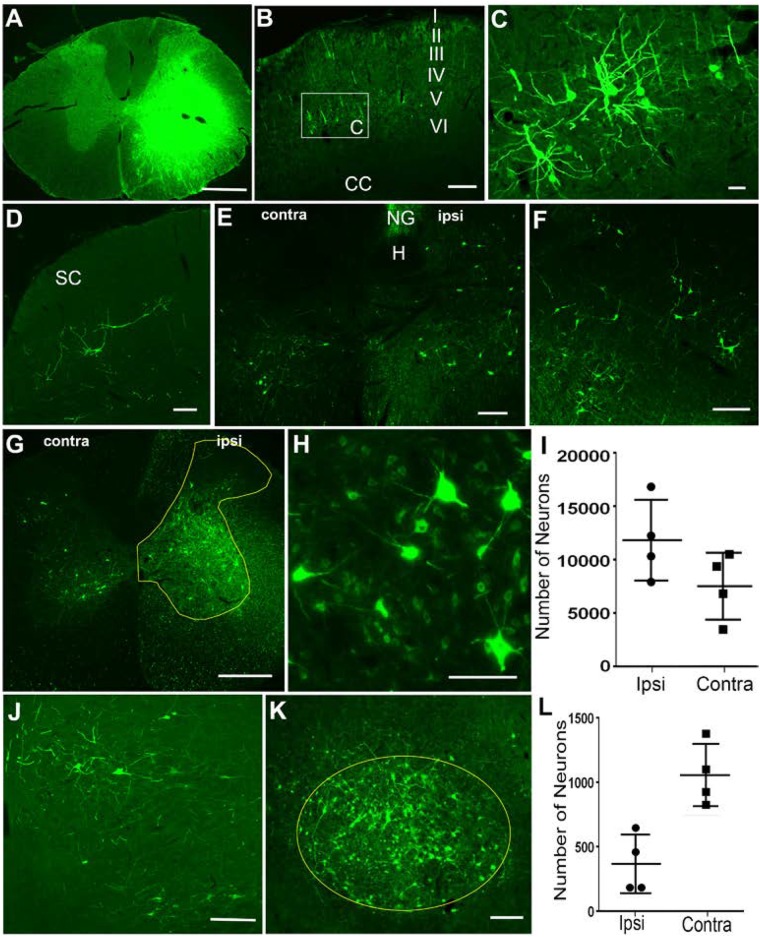
Retrograde labeling of supraspinal and propriospinal neurons after injection of HiRet-GFP into the C6-T1 spinal cord segments. **(A)** Overexposure of GFP labeling at the HiRet-GFP injection site in the C6 spinal cord showing unilateral expression. **(B)** Representative image of contralateral somatomotor cortex showing labeling of layer V pyramidal neurons. **(C)** Higher magnification of box insert of corticospinal neurons from **(B)**. **(D)** Contralateral superior colliculus (SC) with GFP labeled tectospinal neurons. **(E)** Caudal medulla showing bilateral GFP-positive neurons within the ventral part of the medullary reticular formation. Hypoglossal nucleus (H); Nucleus Gracilis (NG). **(F)** GFP-positive neurons labeled within the gigantocellular reticular formation near the ponto-medullary junction. **(G)** Ipsilateral (ipsi) and contralateral (contra) GFP-labeled propriospinal neurons within the C3-C4 spinal cord segments. GFP positive neurons were quantified from entire ipsilateral (or contralateral) gray matter area within the yellow highlighted region. **(H)** Higher magnification of GFP-labeled propriospinal neurons ipsilateral to the injection site. **(I)** Stereological estimates of GFP-positive propriospinal neurons within the C3–C4 spinal cord region showing both ipsilateral and contralateral densities. **(J)** A few GFP-positive rubrospinal neurons were identified within the ipsilateral red nucleus. **(K)** However, the vast majority were localized to the contralateral red nucleus. **(L)** Stereological estimates for the number of GFP-positive rubrospinal neurons identified within either the contralateral or ipsilateral red nucleus. Data is mean ± SD; *n* = 4. Scale bars: **(A,G)**: 500 μm; **(B,D–F,J**,**K)**: 200 μm; **(C**,**H)**: 50 μm.

For this study, we examined the retrograde labeling of neurons synapsing within either the cervical region (C6-T1) or the lumbar region (L1-L4). This cervical region was chosen because it is primarily involved in forelimb motor behaviors such as reaching and grasping; whereas the lumbar region is involved in hindlimb locomotion. Since it is well established that rabies virus is taken up at synapses and transported retrogradely, we used the chimeric rabies-G-envelope protein fused with the cytoplasmic domain of VSV-G. This lentivirus serotype was first developed by Kato et al. (2011b) and shown to induce high efficiency retrograde transport from several neuronal subtypes (Kato et al., 2011a, 2013; Kinoshita et al., 2012; Watakabe et al., 2012; Hirano et al., 2013). HiRet-GFP lentivirus was injected unilaterally into the spinal cord at the designated locations, to map some of the circuits innervating these regions. Examination of the injection site indicated that the virus was confined to the ipsilateral injected side (**Figure 1A**), with occasional labeling of commissural neurons showing a few axons crossing the midline.

In general, GFP-positive neurons were identified in several supraspinal regions known to provide motor information to the spinal cord. Four weeks after C6-T1 HiRet-GFP injections, we observed labeling of cortical neurons in lamina V (**Figures 1B,C**), tectospinal neurons within the deep lamina of the superior colliculus (**Figure 1D**), and various regions in the reticular formation, such as the ventral medullary reticular formation (MdV) bilaterally (**Figure 1E**), and the gigantocellular reticular nucleus (Gi) (**Figure 1F**). This indicates that HiRet-GFP was able to retrogradely label several nuclei of descending motor pathways that are thought to participate in forelimb motor control.

In these studies, we primarily focused on examining GFP-positive neurons localized to either the red nucleus or upper cervical spinal cord. Within the midbrain, neuronal GFP expression was found in the contralateral and ipsilateral red nucleus. The rubrospinal tract (RST) is known to participate in reaching and grasping in rodents as well as monkeys (Van Kan and McCurdy, 2002; Morris et al., 2015; Geed and van Kan, 2017). Rubrospinal neurons were predominately labeled on the contralateral side and GFP-labeled neuronal numbers estimated at 1058 ± 242 neurons using stereological methods (**Figures 1K,L**). Sparse labeling was observed in the ipsilateral red nucleus with 369 ± 227 labeled neurons (**Figures 1J,L**).

Within the spinal cord, C3-C4 PNs contribute to target-directed reaching and hand dexterity by acting as a disynaptic relay between the corticospinal tract (CST) and the C6-T1 forelimb motor neurons in monkeys and cats (Alstermark et al., 1981; Alstermark et al., 1999; Sasaki et al., 2004; Isa et al., 2006; see Alstermark et al., 2007 for review). Injection of HiRet-GFP into the C6-T1 spinal cord segments showed numerous bilateral GFP-positive C3-C4 PNs (**Figure 1G**). Labeled neurons were mostly ipsilateral in the gray matter from Rexed lamina IV to VIII, with somas found both medially and laterally (**Figures 1G,H**). Contralateral GFP expression of PNs was observed mostly medially in the ventral horn in lamina VII and VIII, with sparse neurons observed in the contralateral dorsal horn. Stereological estimates of retrogradely labeled GFP-positive PNs were 12,319 ± 3,853 neurons and 7,927 ± 3,198 neurons for ipsilateral and contralateral C3-C4 gray matter, respectively (**Figure 1I**). The anatomical location and laminar distribution of GFP-positive C3-C4 PNs was identical to retrograde tracing by other methods (Filli et al., 2014; Ni et al., 2014; Han et al., 2015).

**FIGURE 2:**
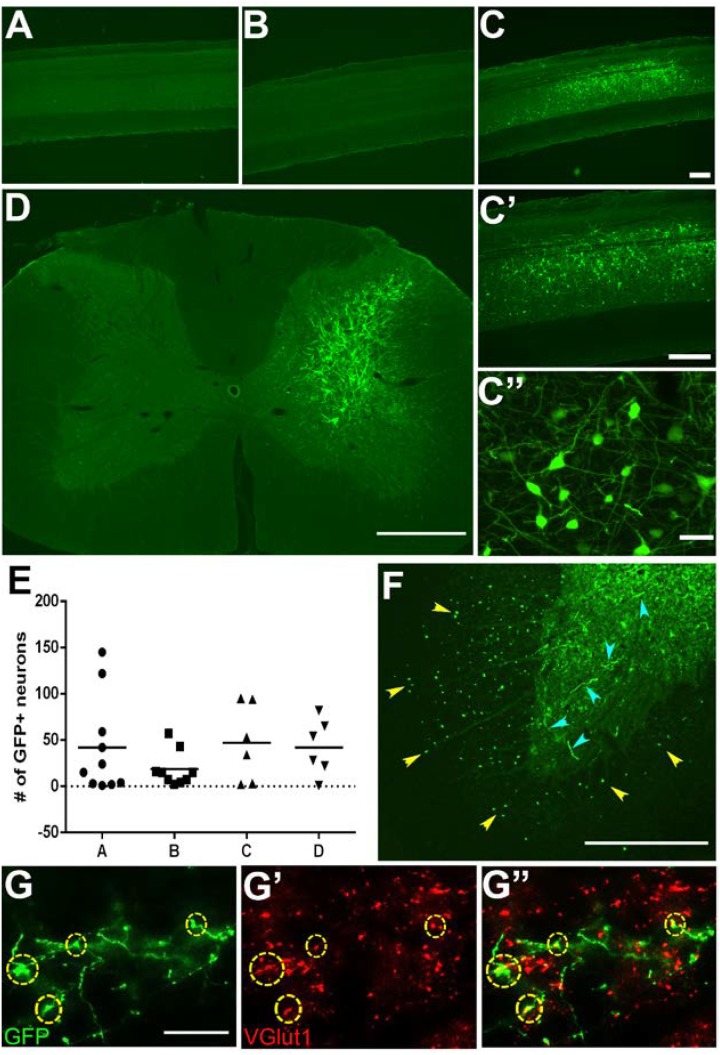
Specific labeling of neuronal subpopulations using HiRet tetracycline-inducible GFP. C3-C4 propriospinal neurons and their synaptic terminals in the C6-T1 spinal cord were labeled by injection of HiRet-TRE-GFP into the C6-T1 and AAV2-TetOn into the C3-C4 spinal cord. Cervical sagittal spinal cord sections from **(A)** AAV2-TetOn control, **(B)** HiRet-TRE-GFP control, **(C)** AAV2-TetOn + HiRet-TRE-GFP following doxycycline administration for 7 days. GFP-positive neurons were identified only in rats receiving injections of both viral vectors with doxycycline. All GFP-labeled neurons were localized to the C3-C4 region where the AAV2-TetOn was injected. **(C’,C”)** Higher magnification images show a cluster of labeled neurons and extensive dendritic arbors. **(D)** Unilateral injections of AAV2-TetOn limited expression of GFP to only the ipsilateral propriospinal neurons. **(E)** All animals showed relatively consistent numbers of GFP-positive neurons labeled per sagittal section (*n* = 4 A–D). This labeling technique allows identification of both the neuronal cell bodies and their axon terminals within the same animal. **(F)** C3–C4 propriospinal axons (yellow arrowheads) within the ventrolateral and ventral funiculi and their axon terminals (cyan arrowheads) within the ventral horn of C7. **(G)** C3–C4 GFP-positive axon terminals within the C7 spinal cord can easily be identified and co-labeled with the pre-synaptic marker vGlut1 **(G’)**, merged **(G”)**. Scale bars: **(A–D)** = 500 μm; **(F)** = 200 μm; **(C”,G)** = 20 μm.

To induce GFP expression within specific neuronal subpopulations we used a two-virus tetracycline-inducible system (Kinoshita et al., 2012). Typically, injections of HiRet-GFP will label multiple populations of neurons innervating a particular target region. Using an inducible promoter limits GFP expression to only the neuronal subpopulation co-expressing the tetracycline transactivating factor (TetOn). We generated HiRet lentivirus designed to drive inducible GFP expression (HiRet-TRE-GFP) and a second adeno-associated virus expressing Tet-On (AAV2-TetOn) to drive inducible expression. The HiRet-TRE-GFP was injected into the C6-T1 spinal cord segments and AAV2-TetOn was unilaterally injected into the C3-C4 spinal cord, allowing specific GFP labeling of only C3-C4 PNs. Four weeks later, rats were injected with doxycycline (30 mg/kg, s.c.) for 1 week. Analysis of spinal cords showed very specific GFP-positive labeling of only the unilateral C3-C4 PNs (**Figures 2C,D**). High density neuronal labeling was observed throughout the cell body and dendrites (**Figures 2C’,C”**), while showing clear consistency in the total number of labeled neurons (**Figure 2E**). Since this method requires two viruses to infect the same cell to induce GFP expression (**Figures 2A,B**), glia at the injection site were not labeled, however, distinct GFP-positive labeling of the axons and their terminals could be identified within the C6-T1 spinal cord (**Figure 2F**). Some of these synaptic terminals co-labeled with vGlut1 (**Figure 2G**). This data indicates that this system can be used to genetically modify specific neuronal populations with synaptic connections within the spinal cord.

### Descending Pathways Into the L1-L4 Spinal Cord

We next wanted to examine pathways involved in hindlimb locomotion. For these studies we injected HiRet-GFP into the L1-L4 spinal cord regions and examined GFP-positive labeling of neurons within the thoracic and cervical spinal cord, as well as various brainstem nuclei, both in the uninjured state and 8 weeks after a severe thoracic contusion.

**FIGURE 3:**
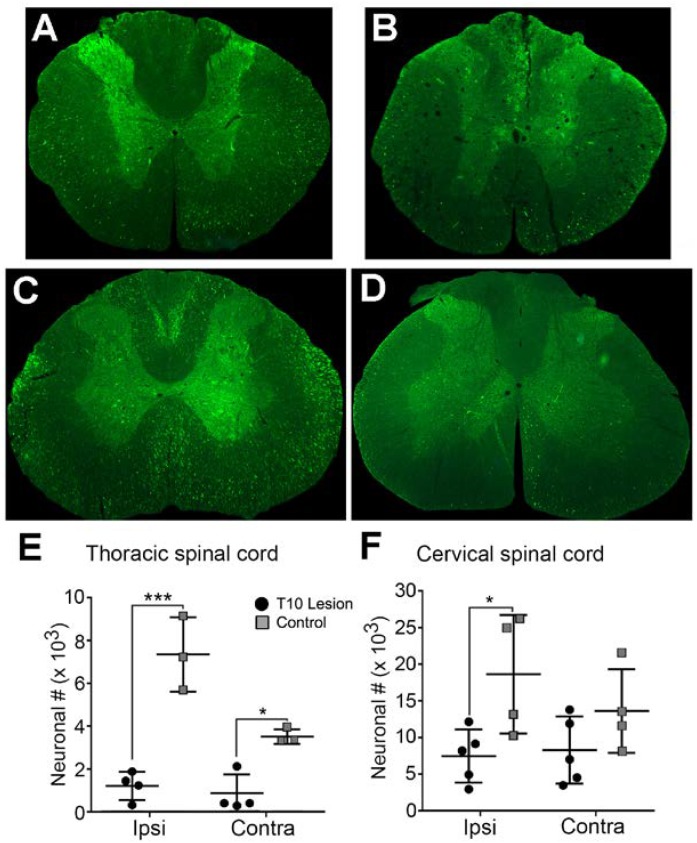
Extent of GFP-expressing neurons within the spinal cord in normal and T10 contused rats. Injection of HiRet-GFP into the non-injured lumbar spinal cord labels many axons and neurons bilaterally within the T7 **(A)** and C4 **(C)** regions of the spinal cord. Injection of HiRet-GFP into the lumbar spinal cord 4 weeks after a 200 kD contusion injury to T10 showed a dramatic reduction in the numbers of axons within white matter tracts and neurons within the gray matter at T7 **(B)** or C4 **(D)** when compared to the GFP-labeling in normal rats. Stereological estimate of HiRet-GFP expressing neurons between the non-injured (control) and 8 weeks post-injury (lesion) shows a significant reduction in the numbers of bilateral thoracic propriospinal neurons **(E)** or ipsilateral cervical neurons **(F)**. Comparisons in both graphs by two-way ANOVA [*F*(1,12) = 9.181, *p* = 0.0105 (cervical) or *F*(1,10) = 68.70, *p* ≤ 0.0001 (thoracic); Sidak multiple comparisons *post hoc* test, *p* = 0.0263 (cervical), *p* < 0.0001 ipsi, *p* = 0.011 contra (thoracic); ^∗^*p* < 0.05, ^∗∗∗^*p* < 0.001]. Data are mean ± SD. *N* = 5 injured, *n* = 4 control. Scale bars: 500 μm.

In the thoracic gray matter, the neuronal population expressing HiRet-GFP in uninjured animals amounted to 7,401 ± 1,744 neurons on the side ipsilateral to the injection, and 3,526 ± 344 neurons in the contralateral cord (**Figure 3A**, quantified in **Figure 3E**). The majority of neurons were clustered in laminae V–VII, though scattered neurons were seen in laminae IV, VIII, IX, and X. Stereological counts 8 weeks after spinal cord contusion showed GFP-positive labeled neurons were reduced to 1,211 ± 653 in the ipsilateral cord and 871 ± 883 in the contralateral cord, with nearly all remaining neurons located in laminae V–VII of the thoracic cord (**Figure 3B**, quantified in **Figure 3E**).

The cervical spinal cord is known to have significant connections with the lumbar spinal cord due to relays between the two central pattern generators (Menetrey et al., 1985; Reed et al., 2006). In agreement with this possibility, we observed a greater proportion of GFP-positive labeled neurons within the cervical cord compared to the thoracic spinal cord. There were 18,777 ± 8,144 neurons seen on the side ipsilateral to the injection and 13,742 ± 5,719 in the contralateral cord in uninjured animals (**Figure 3C**, quantified in **Figure 3F**). As with the thoracic cord, there was a significant loss of GFP-positive labeled neurons in contused animals, though it was significantly less than that observed in the thoracic region. Contusion resulted in an approximately 60% loss (7,572 ± 3,621 neurons) and about a 40% loss (8,407 ± 4,602 neurons) of GFP-positive labeled neurons within the ipsilateral or contralateral spinal cord, respectively (**Figure 3D**, quantified in **Figure 3F**). In uninjured rats, the greatest concentration of neurons occurred in laminae V–VII, with just a few isolated neurons in laminae IX and X; whereas, after contusion the expression was primarily localized to laminae VI and VII, with a few cells in laminae V.

### Connections From the Lumbar Cord to Supraspinal Nuclei

We also analyzed GFP-positive neurons retrogradely labeled in the brainstem, midbrain, and cortex to observe expression in nuclei associated with locomotion, and to analyze connections that may have been rerouted to unexpected areas.

#### Reticular Nuclei

**FIGURE 4:**
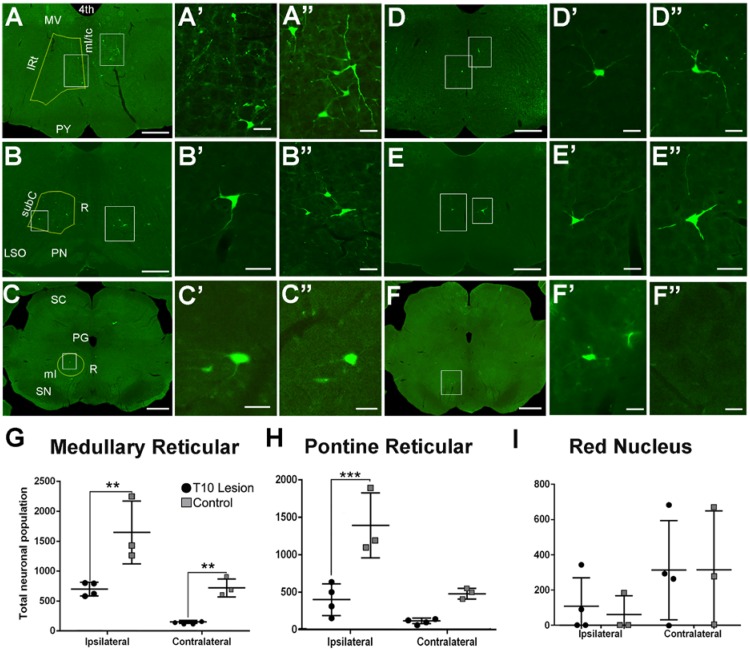
GFP-positive neurons located within the brainstem after injection of HiRet-GFP into the lumbar spinal cord in uninjured and contused rats. Representative images of GFP-expressing neurons within the medullary reticular formation **(A–A”)**, pontine reticular formation **(B–B”)**, and red nucleus **(C–C”)** from HiRet-GFP injections into the lumbar spinal cord. Higher magnification from white boxed inserts from panels A, B, and C showing GFP-positive neurons contralateral **(A’–C’)** or ipsilateral **(A”–C”)** to the injection site. Representative images of GFP-expressing neurons (white boxes) within the medullary reticular formation **(D–D”)**, pontine reticular formation **(E–E”)**, and red nucleus **(F–F”)** from HiRet-GFP injections into the lumbar spinal cord 4 weeks after T10 200kD contusion injury. Higher magnification from respective images showing GFP-positive neurons contralateral **(D’–F’)** or ipsilateral **(D”–F”)** to the injection site. Stereological estimates of neuronal numbers within the medullary reticular formation **(G)**, pontine reticular formation **(H)** and red nucleus **(I)** from non-injured and contused spinal cord. Yellow lines represents regions that were chosen for stereological assessments. Contusion injury significantly reduced the number of GFP-positive neurons within the brainstem. 4th, fourth ventricle; MV, medial vestibular nucleus; IRt, Intermediate Reticular nucleus; ml/tc, medial lemniscus/tectospinal tracts; PY, pyramidal tract; subC, subcoeruleus nucleus; R, raphe; PN, pontine nuclei; SC, superior colliculus; PG, periaqueductal gray; SN, substantia nigra. Comparisons in both graphs by two-way ANOVA [*F*(1,10) = 30.03, *p* = 0.0003 (PRF) or *F*(1,10) = 30.64, *p* = 0.0002 (MRF); Sidak multiple comparisons *post hoc* test *p* = 0.0004 (PRF) or *p* = 0.013 ipsi and 0.0298 contra (MRF) ^∗∗^*p* < 0.01; ^∗∗∗^*p* < 0.001; ^∗^*p* < 0.05]. Data are mean ± SD. *N* = 5 injured, *n* = 4 control. Scale bars: **(A–F)** = 1 mm; **(A’,A”,B’,B”, C’,C”,D’,D”,E’,E”,F’,F”)** = 50 μm.

GFP-positive neurons were localized within several regions of the brainstem and midbrain, most notably the pontine (caudal portion) and medullary reticular nuclei and the red nucleus. In the gigantocellular nucleus of the MRF in uninjured animals, 1,665 ± 532 GFP-positive neurons were labeled on the ipsilateral side, and 728 ± 153 on the contralateral side (**Figure 4A**, quantified in **Figure 4G**). Eight weeks after contusion, GFP-positive neuronal numbers dropped by about 40% to 708 ± 116 on the ipsilateral side and by 80% to 155 ± 24 on the contralateral side (**Figure 4D**, quantified in **Figure 4G**).

A similar density of GFP-positive labeled neurons was found in the caudal pontine reticular nuclei. In uninjured animals, 1,403 ± 433 neurons were labeled on the ipsilateral side, and 492 ± 71 on the contralateral side (**Figure 4B**, quantified in **Figure 4H**). Eight weeks after contusive injury, GFP-positive neuronal numbers decreased 70% to 412 ± 211 neurons on the ipsilateral side and about 75% to 130 ± 36 on the contralateral side (**Figure 4E**, quantified in **Figure 4H**).

#### The Red Nucleus

The red nucleus is a midbrain structure whose total contribution to rodent behavior is not completely clear. Recent evidence from the Whishaw group suggests a role in the arpeggio movement associated with forelimb reaching and grasping, which was hindered when a focused lesion was made in the magnocellular region of the nucleus, the area that gives rise to the RST (Morris et al., 2015). However, the contribution to hindlimb locomotion of the RST is unclear. RST fibers are known to contact the lumbar spinal area, especially in laminae V and VI, and innervate interneuron pools (Holstege and Tan, 1988; Tsukamoto et al., 2003). Thus, we examined the red nucleus for GFP-positive labeling after injection of HiRet-GFP into lumbar spinal cord terminals.

A small population of GFP-positive labeled neurons was seen in the red nucleus in both injured and uninjured animals. RST axons decussate almost immediately upon exiting the red nucleus, and thus more signal can be expected on the side contralateral to the injection site (Liang et al., 2012). In uninjured animals, 315 ± 337 neurons were found on the contralateral side and 61 ± 106 neurons were found on the ipsilateral side (**Figure 4C**, quantified in **Figure 4I**). Interestingly, there was no significant loss of neurons seen in contused animals, with 314 ± 283 neurons seen on the contralateral side, and 109 ± 163 neurons quantified on the ipsilateral side (**Figure 4F**, quantified in **Figure 4I**).

## Discussion

Unlike traditional retrograde tracers, viral retrograde tracers allow genetic manipulation of neurons that innervate a specific region of the spinal cord. Here, we show that discrete unilateral injections of HiRet lentivirus into either the cervical or lumbar spinal cord label well-defined locations within the spinal cord and brainstem when compared to previous studies using retrograde chemical tracers. This labeling is found to be stable *in vivo* for extended time periods (we have observed GFP expression for up to 4 months), making it superior to tracers such as BDA or pseudo-rabies virus, which must be dealt with in a time-sensitive manner. Additionally, we have found no evidence of neuronal cell death in either the brain or spinal cord while using this tracer. HiRet lentivirus, however, substitutes the VSV-g for a rabies G/VSV-g chimeric envelope glycoprotein to enable preferential uptake at synaptic terminals, unlike traditional chemical retrograde tracers that can be taken up by damaged axons or axons en passage (see Kobayashi et al., 2016 for review). In this regard, we have observed no neuronal labeling of spinal motor neurons or DRG neurons after injection of HiRet-GFP into the transiently demyelinated sciatic nerve, however, injection of AAV2-mCherry shows very good labeling of both populations. Thus, this viral vector supports better definition of pathways that innervate specific regions of the brain or spinal cord and provides an advantage compared to AAVs that are used for retrograde mapping (e.g., rAAV2-retro) where uptake by axons en passage needs to be taken into account (Tervo et al., 2016). We have also found no evidence of *trans*-synaptic transport by this vector. For example, we observed no GFP labeling of neuronal populations that do not make direct synaptic connections into either the cervical or lumbar region even after increasing injection volume or waiting for longer time periods after injections. These advantages could prove critical not only for mapping of circuits from the brain to the spinal cord, but for studies specifically targeting neuronal populations for silencing or ablation (Kinoshita et al., 2012; Liu et al., 2017; Tohyama et al., 2017).

In the present study, we examined the utility of a retrogradely transportable lentivirus to map pathways terminating within either the cervical or lumbar spinal cord. We observed very good retrograde transport into multiple pathways known to be involved in motor control, primarily within the brainstem and spinal cord. However, retrograde labeling of cortical motor neurons was relatively weak, so this method might not be as useful for that pathway. The number and distribution of neurons retrogradely labeled using HiRet lentivirus compared well to those labeled using other methods (e.g., fluorogold, microruby) (Reed et al., 2006; Liang et al., 2011). For example, fluorogold tracer injected into multiple mouse cervical spinal cord segments showed average ipsilateral and contralateral neuronal counts of 1,337 and 3,612 for the red nucleus, 1,001 and 532 for the caudal part of the pontine nucleus, and 2,744 and 1,148 for the ventral MRF (Liang et al., 2011). These numbers are comparable to those in the present study considering our injections are localized to specific regions and from either the cervical or lumbar spinal cord. Unfortunately, the retrograde labeling of the cortical motor neurons was relatively weak, in which we observed labeling of about a tenth of those reported previously (Liang et al., 2011). Thus, this virus method would not be as useful for the corticospinal pathway. Finally, this viral tracer can be used to map changes in intraspinal and supraspinal circuit pathways pre- and post-SCI, to highlight neuronal populations potentially involved in spontaneous behavioral recovery or contributing to functional recovery in experimental models.

The cervical region of the spinal cord contains the spinal motor neurons for forelimb movements. The CST is considered the primary pathway for control of skilled forelimb reaching and grasping and we observed some, but minor, labeling of this pathway after cervical injections of HiRet-GFP. However, the CST also sends numerous collateral branches into the brainstem nuclei and data in monkeys and cats have identified an important role of C3-C4 propriospinal neurons in forelimb patterning (Alstermark et al., 1981, 1999; Sasaki et al., 2004; Isa et al., 2006; Kinoshita et al., 2012; see Alstermark et al., 2007 for review). For this reason, we were primarily interested in labeling C3-C4 propriospinal neurons since several studies in the cat and monkey have demonstrated the importance of the C3-C4 propriospinal pathway in the recovery of reaching and grasping after CST lesions (see reviews by Flynn et al., 2011; Alstermark and Isa, 2012). We observed very robust bilateral labeling of these propriospinal neurons, showing much better details of dendrites than chemical tracers, which mostly only label the cell bodies. Cervical injections of HiRet with GFP under the control of the tetracycline-inducible promoter showed very nice unilateral expression, since AAV encoding TetOn was only injected into the right side of the spinal cord. This two vector inducible approach, first described by Kinoshita et al. (2012), supports the tracing of the entire labeled neuron from the dendrites to the axonal terminals within the C6-T1 spinal cord region. In addition to the C3-C4 propriospinal neurons, several studies have also demonstrated the red nucleus as an important midbrain structure potentially involved in forelimb reaching and grasping after CST lesions (Whishaw et al., 1990; van Kan and McCurdy, 2001; Horn et al., 2002; Kuchler et al., 2002), and after bilateral pyramidotomy, CST axons are known to sprout extensively into the red nucleus and to promote recovery of skilled forelimb function (Mosberger et al., 2017). Discrete injections of HiRet-GFP into the C6-T1 spinal cord showed robust labeling of rubrospinal neurons, a potential target for behavioral studies examining forelimb function post-spinal cord injury.

The lumbar cord contains both the central pattern generator and motor neurons that connect to the muscles of the hindlimb involved with locomotion (Rossignol, 2000). Thoracic contused animals initially lose the ability to step and bear weight with their hindlimbs after a moderately severe (200 kilodyne) injury, but spontaneously recover some stepping without coordination by about 4 weeks (Scheff et al., 2003). Spontaneous recovery of hindlimb locomotion often plateaus at this stage, and it is reasonable to assume that plasticity of spared neuronal circuits may contribute and compensate for lost supraspinal input. Application of HiRet-GFP to the area below the lesion allows neurons with synaptic terminals in this area to uptake the virus and express GFP, potentially labeling neuronal populations important to behavioral improvement.

Following injection into the lumbar spinal cord, HiRet-GFP labeled propriospinal interneurons in the cervical and thoracic spinal cord, red nucleus, and discrete reticular and vestibular nuclei in the brainstem. Although we observed few GFP-positive neurons within the somatomotor cortex or red nucleus, we did observe high numbers of neurons within several regions of the reticular formation. The reticular formation consists of a large cluster of nuclei in the brainstem known to play an important role in locomotion. These fibers run in two main bundles – the medial reticular spinal tract, which originates in the PRF of the pons, and the lateral reticular spinal tract, which originates in the MRF of the medulla. Within the MRF, the nucleus reticularis gigantocellularis is known for integration of motor commands from the mesencephalic motor region and other areas (Filli et al., 2014; Zorner et al., 2014). In normal rat brains, we observed fairly robust labeling of neurons in the caudal part of the PRF and the gigantocellular portion of the medulla. After spinal cord contusion the numbers of GFP-positive neurons within the reticular formation decreased dramatically. Although the majority of connections are lost after injury, the reticulospinal fibers have been associated with spontaneous recovery by compensatory sprouting (Ballermann and Fouad, 2006; Filli et al., 2014; Zorner et al., 2014), particularly onto propriospinal neurons that send their axons within the lateral and ventral funiculi, which remains partially intact after a contusive injury.

Numerous studies have indicated that recovery of hindlimb locomotion after spinal cord injury is mediated by sprouting of supraspinal axons onto propriospinal neurons bypassing the lesion (Bareyre et al., 2004; Courtine et al., 2008; van den Brand et al., 2012). As with some reticular axons, these axons travel primarily within the ventral lateral and ventral white matter of the spinal cord. Injections of HiRet-GFP into the lumbar cord of uninjured rats showed dense labeling of both cervical and TPNs. We focused on two types of PNs in this study: the short descending thoracic propriospinal interneurons (TPNs) that connect the thoracic and lumbar segments and the long descending propriospinal interneurons (LDPNs), which connect the cervical to lumbar regions (Sherrington and Laslett, 1903; Kostyuk and Vasilenko, 1979; Chung and Coggeshall, 1983). The latter group is thought to synchronize activity between the cervical and lumbar central pattern generators (Miller et al., 1973; Zaporozhets et al., 2006). Depending on the lesion type or location either propriospinal population has been shown to contribute to recovery of locomotion (Bareyre et al., 2004; Courtine et al., 2008; van den Brand et al., 2012; Filli et al., 2014), indicating that either population could act as a relay to send motor commands past a spinal cord lesion. In our study, we observed a greater loss of GFP-positive labeled propriospinal neurons within the thoracic compared to the cervical spinal cord. The contusion injury model used may cause higher levels of cell death within thoracic neurons due to their proximity to the lesion site. Indeed, TPNs undergo increased cell death and show higher levels of apoptotic markers after thoracic injury (Siebert et al., 2010a,b; Conta Steencken et al., 2011). The relatively low amount of expression that we saw in TPNs after injury could indicate that in our lesion model, spontaneous recovery is more dependent on the cervical population of LDPNs.

## Conclusion

HiRet lentivirus provides us with the ability to label neuronal populations within the brain and spinal cord with high fidelity. This viral vector can be utilized as a retrograde tracer to map pathways pre- and post-injury and to target specific neuronal populations. HiRet permits stable, long lasting transgene expression, providing a significant advantage over chemical tracers and neurotoxic retrograde viruses. In addition, this vector may allow detailed and specific analysis of interneuronal circuits that undergo plasticity post-injury. When combined with vectors allowing tetracycline-inducible transgene expression, these pathways are viable targets for investigating functional recovery.

## Author Contributions

IS, KK, and GS: wrote paper text, contributed images, and conceived and designed the experiments. IS, KK, NS, IJ, CE, YL, and X-QT: performed the experiments and analyzed the data.

## Conflict of Interest Statement

The authors declare that the research was conducted in the absence of any commercial or financial relationships that could be construed as a potential conflict of interest.
